# Assessment of Differential Perceptions of Core Nursing Competencies between Nurse Managers and Nursing Graduates: A Cross-Sectional Study

**DOI:** 10.3390/nursrep13040145

**Published:** 2023-12-18

**Authors:** Mahdi Tarabeih, Wasef Na’amnih

**Affiliations:** 1 School of Nursing Sciences, The Academic College of Tel-Aviv-Yaffa, Tel Aviv 64044, Israel; wasefna@mta.ac.il; 2 Department of Epidemiology and Preventive Medicine, School of Public Health, Sackler Faculty of Medicine, Tel Aviv University, Tel Aviv 69978, Israel

**Keywords:** competence, nursing education, nursing skills

## Abstract

Background: The literature review on perceptions of nursing competencies shows a critical shortage of studies addressing this topic. Aim: To examine and compare perceptions of important nursing competencies of nursing graduates, and nurse managers. Methods: A cross-sectional study was conducted among 148 students, who were recent graduates of the nursing school with RN degrees and had completed advanced training lasting 3 months at the Sheba Academic School of Nursing and the Academic School of Tel Aviv-Yafo, and 183 nurse managers with at least one year of seniority in the position in hospitals and community clinics in Israel. The recruitment and data collection of the nurse managers lasted 12 months, from July 2021 to July 2022, and for nursing graduates, two months, from June 2022 to July 2022. An online questionnaire was constructed and validated by five professional and experienced individuals in the research domains for adequate content validity. The questionnaire consisted of 47 items in total. Domains included: (1) professionalism in research; (2) skills for direct treatment; (3) support and communication; (4) professional knowledge; (5) personal abilities; and (6) critical thinking and innovation. Results: Overall, 331 valid questionnaires were collected (85% response rate). The difference in the nurse’s research professionalism index was found to be statistically significant with a higher rating given by nurse managers, *p* = 0.003. However, the difference in the direct treatment index of nurses was found to be statistically not significant, *p* = 0.610, between newly graduated nurses and nurse managers. The independent sample *t*-test indicated a significant difference with a higher rating among nurse managers in the nurse’s support and communication index, *p* = 0.020; professional knowledge index, *p* < 0.001; personal abilities index, *p* < 0.001; and critical thinking index, *p* = 0.006, between newly graduated nurses and nurse managers. Conclusion: Nursing education programs aimed at training future nurses with core competences should include a curriculum designed to promote the acquisition of these six core competences leading to a congruence between the role expectations of nurse managers and those of newly graduated nurses.

## 1. Introduction

In times of increasing pressure to meet the demand for healthcare services, newly graduated nurses constitute an important cohort of the nursing community and an important human resource, bringing fresh vigor to the healthcare environment [1]. Enhancing the successful transition from school to practice is imperative to retaining them in the workforce [2] as well as to providing high-quality healthcare. The World Health Organization attributed the poor quality of healthcare to a lack of healthcare provider competence. An increase in life expectancy and rapid advances in technology, medical sciences, and the healthcare system have contributed to the rise in both standard of living and life expectancy, leading to greater demands for quality health services. The contribution of nurses to the quality of care for patients and their families has been found in many studies to be perceived as more significant than the contribution of their medical colleagues in a multi-disciplinary team. Despite the growing demand for nurses to possess high-quality competences, skills, and personal abilities in order to manage chronic diseases and treatment of terminal patients, studies show that there is a gap between the training they receive in professional schools and what is required of them in the field [3]. There is a close connection between maintaining a high level of competency and the ability to offer patients high-quality healthcare [4]. In the years 2001 and 2009, the World Health Organization published two reports entitled ‘Global Standards for the Initial Education of Professional Nurses and Midwives’ with the aim of defining competence requirements for the core education of nurses [5,6].

As healthcare becomes increasingly complex, it is imperative to reduce adverse outcomes by ensuring the delivery of safe, quality care to patients. Nursing faces a challenge in determining how to maintain competence to provide an optimal level of care [7]. Understanding the core competences and continuously evaluating the competence levels of nurses is likely to influence standards of care [8,9]. Also, assessment of competence in nurses helps in determining professional development needs and areas for quality improvement [10]. Failure to monitor competence levels is postulated to have an adverse effect on the quality and safety of care [11].

Through simulations, mentorships, competency assessments, and clinical practice, graduating nurses leave schools with a certain degree of professional self-efficacy. The competence of practicing nurses is a requirement of accreditation bodies and a standard of practice of the American Nurses Association [4]. The recent reforms of educational systems within industrialized nations have moved nursing education away from process-based training programs to competency-based training programs [12]. Nursing practice requires a complex combination of various attributes; this requirement is reflected in one of the first definitions of nursing competence, formulated by Short [13]: “The ability of the registered nurse to integrate and apply the knowledge, skills, judgments and personal attributes required to practice safely and ethically in a designated role and setting”. This was a widespread definition of competence specified by 10 different nursing regulatory bodies in Canada [14].

Several concepts and definitions of competence and competency are presented, including those from nursing regulatory bodies in Canada, the American Academy of Ambulatory Nursing, and other generalized definitions. These definitions revolve around the integration and application of knowledge, skills, judgments, and personal attributes necessary for safe and ethical practice. The American Academy of Ambulatory Nursing defines competence as “having the ability to demonstrate the technical, critical thinking, and interpersonal skills necessary to perform one’s job” [15]. Many institutions of healthcare higher education throughout the world have developed or adapted to harmonized nursing training programs with the goal of ensuring the acquisition of defined nursing competencies [16]. Foss et al. defined these as the amalgamation of abilities, knowledge, and skills essential for the execution of a specific task [17]. Within the realm of nursing, competency encapsulates the profound mastery of a pertinent body of knowledge, complemented by a spectrum of skills, which span clinical, interpersonal, and technical dimensions, vital for efficacious performance in a clinical setting [18]. Competence-based education delineates the requisite criteria that learners must meet to evince competency [19]. This educational approach necessitates the pinpointing of fundamental competencies indispensable for delivering holistic nursing care in clinical contexts. The common understanding is that competence is required to apply the knowledge from the studies, to interact with the patients and the staff, and to provide quality care. Thus, the rationale for our study is the increasing demand for competent graduates and the need to develop Israeli national standards of core competences and develop a psychometric instrument to identify and measure the core competencies needed by undergraduate nursing graduates.

Advancements in nursing training programs serve as a basis for expanding the nurses’ authority and increasing the areas of responsibility assigned to the nursing staff. This trend of professional development is supported by the perception that nurses with a high level of professional training and academic education will provide a better standard of care. The growth and aging of the population and the multiplicity of patients with multimorbidity and complex needs have combined with the trend in professional development to create a demand for more efficient training of nurses aimed at producing graduates with a high standard of professional qualifications. These include proficiency in handling information and learning skills, critical thinking, communication skills for counseling and support, technical skills, moral competence [20], and commitment to continued learning. All of this is required by a healthcare system that provides care at a high standard and fosters young leadership. Therefore, our vision for the future is to tailor the curriculum to the workplace. This is because the congruence between the ratings given by the graduates and the nurse managers of the importance of the six core nursing competencies is based on the advanced clinical practice period experienced by the young graduates. Today, there is a demand for nursing educators to provide learning opportunities and to train nurses in medical science technology and the health system who have the potential for young leadership to bridge the gap between newly graduated nurses and nurse managers, and to minimize the discrepancy between the training received in professional schools and what is required in the clinical field to ensure nursing competency. A good alignment between the perceived necessary skills held by nurse managers and those of the new nursing graduates should lead to job satisfaction and occupational commitment of the young nurses, reducing attrition from the profession or from work that meets international standards.

The definition of competency refers to the ability to apply learned knowledge and treatment skills, to interact with patients and colleagues, to make decisions in a changing reality, and to provide quality care [21]. In the USA and Europe, there are reports of a gap between the graduates of nursing training programs and the minimum standard required for professional competency [22], i.e., according to Institute of Medicine Reports [23], 49% of new graduate nurses were involved in safety violations during the treatment of patients; 75% of these were medication errors. Researchers in Australia [24] and China [25] attest that new graduate nurses have difficulty functioning in a dynamic and constantly changing healthcare environment. In order to ensure nurses’ competence, it is vital to continue to measure the outcomes of nursing program graduates using competence assessment tools [4].

Several studies have found that new graduate nurses lack adequate preparation for entering the workforce and fail to meet the demands of the contemporary healthcare environment [26,27,28]. Others have stated that educational programs do not adequately prepare new graduate nurses for the real life of nursing practice. The perception that new graduate nurses have low levels of clinical competence has created tension between nurse educators and nurses working in the field [29]. Thus, it is imperative to have an understanding of graduating nurses’ perceptions of nursing competence.

The literature review on perceptions of nursing competencies shows a critical shortage of studies addressing this topic. In order to fill this gap, we performed a study among nursing graduates and nurse managers. The purpose of our study is to examine and compare the perceptions of important nursing competencies as rated by nursing graduates, and nurse managers. Gaps and differences in perceptions of these two groups may indicate an incompatibility between training programs and nursing work in actual practice. The research questions that guided our study are:

RQ1: What are the nursing competencies that are perceived as highly important by nurse managers and by graduating nursing students?

RQ2: Do nurse managers and graduating nursing students perceive the importance of the various nursing competencies in a similar manner?

## 2. Materials and Methods

### 2.1. Study Design and Setting

A cross-sectional study was conducted among students who were recent nursing school graduates granted the RN degree and had completed advanced training lasting three months at the Sheba Academic School of Nursing and the Academic School of Tel Aviv-Yafo and among nurse managers with at least one year of seniority in the position in hospitals and community clinics in Israel. The recruitment and data collection of the nurse managers lasted 12 months, from July 2021 to July 2022, and for nursing graduates, 2 months, from June 2022 to July 2022.

### 2.2. Study Measure and Participants

The study measure is an internationally validated questionnaire. The validation was conducted for each of the six domains in the questionnaire. The reliability of this instrument was examined and found suitable for measuring the profile characteristics of a graduate nurse with professional training in an academic program [25]. We used the psychometric measurement instrument [25], which is designed to examine the psycho-technical competencies of the graduates and their suitability to the requirements of the demanding health system. The online questionnaire was created using the Qualtrics method, professionally translated from English to Hebrew, constructed and validated by five professional and experienced individuals in the field of research for adequate content validity. The questionnaire consisted of 47 items in total and was addressed specifically to 390 Israeli nurses by a link to the questionnaire. Of those, 331 nurses completed the questionnaire in full (a response rate of 87%). In total, 148 of them were nursing graduates (71 women, 48%), with a mean age of 28.14 years and a standard deviation (SD) of 5.62. Additionally, 183 out of 220 nurse managers in hospitals and the community, who had been in a managerial position for at least a year (159 women, 87%), with a mean age of 43.44 years (SD = 10.31), completed the questionnaire, with a response rate of 83%. Sixty-nine questionnaires were disqualified due to significant missing data. A pilot study was conducted with 30 nurses, resulting in a Cronbach’s alpha of 0.78 for all the questions in the questionnaire. Items were presented on a five-point Likert scale in which 1 denotes ‘definitely not important’; 2 denotes ‘not important’; 3 denotes ‘neutral’; 4 denotes ‘important’; and 5 denotes ‘definitely important’. In this way, we avoided potential bias by presenting two responses that reflect lower importance and two responses that reflect high importance. The questionnaire consisted of two parts. The first part included demographic information (age, gender), advanced training, and job seniority. The second part addressed the following six domains: (1) professionalism in research—research capability, writing skills (in English as a research language), and digital information skills (internal reliability: Cronbach’s alpha 0.844); (2) direct patient care—performing basic and advanced nursing skills safely and securely (internal reliability: Cronbach’s alpha 0.877); (3) support and communication skills—emphasizing effective communication skills with patients and enhancing their well-being through social and emotional support (internal reliability: Cronbach’s alpha 0.853); (4) professional knowledge—relevant clinical knowledge, managing nursing care for patients, quality of care, and health promotion (internal reliability: Cronbach’s alpha 0.855); (5) personal abilities—self-confidence, self-control, and professional competency (internal reliability: Cronbach’s alpha 0.822); (6) intellectual skills—valuing critical thinking, self-assessment, and personal commitment to continuous learning and innovation (internal reliability: Cronbach’s alpha 0.733) (Supplementary Table S1).

### 2.3. Data Analysis

Factor analysis was used to build scores. Cronbach’s alpha was used to assess internal consistency across the items that were included in these scales. The assumption of the normal distribution was performed using the Kolmogorov–Smirnov test for the study variables (domains). Variables in our study were distributed normally and described as the mean and SD using Student’s *t*-test. All statistical tests were two-sided, and *p* < 0.05 was considered statistically significant. Data analysis was performed using Statistical Package for the Social Science (SPSS) version 28 (IBM, Armonk, New York, NY, USA).

### 2.4. Ethics Approval

The study protocol was approved by the Institutional Review Board (IRB) at the Academic College of Tel Aviv-Yaffo (Protocol number 2021-1093); all procedures were performed in accordance with local guidelines and regulations. The questionnaire was filled out anonymously. Informed consent was obtained from nurses before participation in the study.

## 3. Results

Overall, 331 valid questionnaires were collected (85% response rate). In order to answer RQ1, we calculated the descriptive measures of each of the six domains. Table 1 presents the means and SD of the importance attached to each domain. All of the six competencies were rated as highly important to the work required of competent nurses and ranged from 3.99 to 4.46 on a five-point Likert scale. The least important competency as rated by respondents is the personal abilities index, where the nurse managers’ mean rating is 4.38 (SD = 0.52) and the newly graduated nurses’ mean rating is 3.99 (SD = 0.78). The most important competency as rated by respondents is the direct treatment index, where the nurse managers’ mean rating is 4.54 (SD = 0.41) and the newly graduated nurses’ mean rating is 4.57 (SD = 0.55).

In order to answer RQ2, we calculated a *t*-test for independent samples with the aim of detecting any differences between the rating of each of the six domains given by the nurse managers and those of the newly graduated nurses. Although all *t*-tests indicated significant differences, with the nurse managers’ ratings being higher than those of the newly graduated nurses, these differences are very small and have no theoretical meaning. See *t*-test values in Table 1.

An independent sample *t*-test was calculated in order to examine the differences in six domains between newly graduated nurses and nurse managers. The difference in the nurse’s research professionalism index was found to be statistically significant with a higher rating given by nurse managers, *t* = −2.971, *p* = 0.003. However, the difference in the direct care index of nurses was found to be statistically not significant, *t* = 0.492, *p* = 0.610, between newly graduated nurses and nurse managers. The difference in the support and communication index of nurses was found to be significant with a higher rating among nurse managers, *t* = −2.251, *p* = 0.020, between newly graduated nurses and nurse managers.

The independent sample *t*-test indicated a significant difference with a higher rating given by nurse managers in the nurse’s professional knowledge index, *t* = −5.495, *p* < 0.001; personal abilities index, *t* = −5.294, *p* < 0.001; and critical thinking index, *t* = −2.710, *p* = 0.006, between newly graduated nurses and nurse managers (Table 1).

## 4. Discussion

The current study sought to reveal the nursing competencies that are perceived as important by newly graduating nurses. We also examined whether there were differences between the importance attached to these nursing competencies when rated by newly graduated nurses as compared to nurse managers. All of the six competencies were rated as highly important to the work required of competent nurses and ranged from 3.99 to 4.46 on a five-point Likert scale. We compared the importance attached to each competency by nurse managers versus graduating nursing students and found that the nurse managers’ ratings were higher than those of the newly graduated nurses with regard to the research professionalism index, support and communication index, professional knowledge index, personal abilities index, and critical thinking index; the difference regarding the direct care index of nurses was found to be statistically insignificant. These findings are generalizable to similar contexts of other nursing training programs and nurse managers in medical centers.

Based on their experience, nurse managers know what skills and abilities are important for the proper professional functioning of nurses in the field. Congruence between the perceptions of the required competencies held by the nurse managers and those of the newly graduated nurses should lead to job satisfaction and occupational commitment of the young nurses. Without such agreement, the new nurses may have a higher intention to leave the profession during the first year after graduation [1].

The research professionalism competence of nurses is important to their performance due to its impact on the clinical care they provide. Nurses with a passion for nursing research may seek to improve their critical thinking and research dispositions. Nurse educators should therefore provide better learning, working, and research opportunities [30]. Critical thinking is an important nursing competence and has been found to be a skill that characterizes experienced RNs [31]. Innovation has been consistently highlighted as an effective factor in facilitating efforts for the advancement of healthcare quality [32] because it facilitates the generation or implementation of new ideas. Support and communication skills are crucial to the ability to provide effective treatment; the nursing profession requires an ability to interact with colleagues as well as with patients and their families [33]. Inadequate communication skills can be very problematic in the clinical setting, as it may increase the risk of miscommunication. The ability to provide direct care to the patient is consistently evaluated as important and crucial because it directly impacts the quality of patient care [25]. Competent nurses should ideally possess certain personal abilities such as self-confidence and self-control, yet such abilities that are necessary for working in some fields of nursing are not taught as part of undergraduate education [34].

The literature on nursing education and training shows a wide consensus regarding the difficulties surrounding the transition from being a nursing student to a beginner qualified nurse; this transition involves many significant professional and personal challenges [2,35,36,37,38,39]. The learning process for newly graduated nurses during their transition to the nursing profession is stressful and challenging [40]. This transition process is sometimes referred to as ‘reality shock’ [41]. Reality shock is a term used to describe the reaction of an individual who has received education in a professional field and has just started working [36,38,41]. Kramer et al. defined reality shock as “the reactions of new workers when they find themselves in a work situation for which they have spent several years preparing and for which they thought they were going to be prepared, and then suddenly find they are not” [38].

Contemporary views of the professional socialization of nurses recognize the crucial role of the personal values that are associated with a profession. Fagermoen [42] emphasized that values and beliefs play a central role in shaping the professional identity of nurses, meaning that a nurse’s professional identity is defined by her philosophy of nursing. Hence, several studies have pointed to potential reality shock for newly graduated nurses stemming from discrepant role conceptions [38]. Dyess and Sherman [37] claim that there is evidence to suggest that the problems associated with the transition into nursing practice are more serious today than in the past. Our findings indicate the opposite and imply that with a proper curriculum, the transition might be experienced more easily by newly graduated nurses. The findings of the present study show that newly graduated nursing students understand their role quite similarly to the way nursing is understood by experienced nurse managers. We found that they evaluate the required level of knowledge and mastery of clinical and communication skills, as well as an appreciation for critical thinking and research professionalism in a manner similar to the nurse managers. We believe that the congruence between the graduates’ and the nurse managers’ ratings of the importance of the six core nursing competencies stems from the advanced clinical practice period experienced by the young graduates. It is known that extended, significant clinical experience leads to better assimilation of knowledge and skills as well as familiarity with norms as a basis for professional socialization [43]. Hence, it is very likely that the newly graduated respondents underwent a socialization to the nursing profession during their advanced practice period that led them to evaluate the importance of the six domains of competencies similarly to the way that nurse managers do [44].

### Limitations of the Study

Our study had some limitations. The generalizability of the findings and conclusions drawn in the current study is restricted to a comprehensive three or four-year curriculum. However, the conclusions may not be generalizable to shortened programs which do not include all of the courses that train for proficiency in the six domains that were studied, such as communication skills, management and leadership, professionalism in research, and direct care to patients.

The information was obtained by self-reporting from the participants, which might have led to non-differential misclassification. However, there is no other way to obtain information regarding evaluating competence other than self-reporting by the participants, since such information is usually not available in administrative databases. In addition, in this type of variable, the personal perception of the participants is important; thus, the self-report on evaluating competence does not necessarily constitute a limitation. The premise of this study is that students’ perceptions of the importance of each of the six measured competencies reflect their preparedness for work in the field. However, it should be borne in mind that the relationship between the perceptions and the level of actual performance was not directly examined in the current study.

It would be worthwhile to examine how students’ perceptions change over the years of schooling by comparing the perceptions of first-year students to those of second, third, and fourth-year students. Also, we believe that a qualitative study of the challenges experienced by newly graduated nurses would inform decisions relating to some potential changes required in nursing education programs.

## 5. Conclusions

Our study findings suggest that nursing education programs should include adequate educational curricula aimed at fostering the following six core competencies which are considered highly important: research professionalism; skills for direct treatment; support and communication skills; professional knowledge; personal abilities; and critical thinking. Nursing education programs aimed at training future nurses with core competencies should include an adequate curriculum designed to promote the acquisition of these six core competencies leading to a congruence between the role expectations of nurse managers and those of newly graduated nurses.

## Figures and Tables

**Table 1:** Importance and relevance of nursing competencies, comparing newly graduated nurses and nurse managers.

Domain	Newly Graduated Nurses (N = 148)		Nurse Managers (N = 183)		Significance	
	Mean	SD	Mean	SD	*t*-Test	*p*
**Research Professionalism**	4.30	0.52	4.46	0.42	−2.971	0.003
**Direct Treatment**	4.57	0.55	4.54	0.41	0.492	0.610
**Support and Communication**	4.27	0.63	4.42	0.47	−2.251	0.020
**Professional Knowledge**	4.00	0.70	4.40	0.55	−5.495	<0.001
**Personal Abilities**	3.99	0.78	4.38	0.52	−5.294	<0.001
**Critical Thinking and Innovation**	4.20	0.81	4.42	0.58	−2.710	0.006

## Data Availability

The data presented in this study are available on request from the corresponding author.

## Supplementary Materials

The following supporting information can be downloaded at: https://www.mdpi.com/article/10.3390/nursrep13040145/s1, Table S1: Scales for evaluating study variables in factor analysis.

## Public Involvement Statement

There was no public involvement in any aspect of this research.

## Guidelines and Standards Statement

This manuscript was drafted against the Strengthening the Reporting of Observational Studies in Epidemiology (STROBE) for the quantitative study and Consolidated Criteria for Reporting Qualitative Research (COREQ) for the qualitative study.

## References

[1] Murray M., Sundin D., Cope V. (2019). Benner’s model and Duchscher’s theory: Providing the framework for understanding new graduate nurses’ transition to practice. Nurse Educ. Pract..

[2] Regan S., Wong C., Laschinger H.K., Cummings G., Leiter M., MacPhee M., Rhéaume A., Ritchie J.A., Wolff A.C., Jeffs L. (2017). Starting out: Qualitative perspectives of new graduate nurses and nurse leaders on transition to practice. J. Nurs. Manag..

[3] Lee S., Perry F. (2018). Supporting transition milestones in the nursing curricula. J. Nurs. Educ..

[4] Church C.D. (2016). Defining competence in nursing and its relevance to quality care. J. Nurses Prof. Dev..

[5] World Health Organization (2001). Global Advisory Group on Nursing and Midwifery. https://www.who.int/hrh/resources/28NOV13008_GlobalAdvisoryGroupNursingMidwifery.pdf?ua=1.

[6] World Health Organization (2009). Global Standards for the Education of Professional Nurses and Midwives. https://apps.who.int/iris/bitstream/handle/10665/44100/WHO_HRH_HPN_08.6_eng.pdf.

[7] Smith S.A. (2012). Nurse competence: A concept analysis. Int. J. Nurs. Knowl..

[8] Axley L. (2008). Competence: A concept analysis. Nurs. Forum.

[9] Tabari-Khomeiran R., Kiger A., Parsa-Yekta Z., Ahmadi F. (2007). Competence development among nurses: The process of constant interaction. J. Contin. Educ. Nurs..

[10] Meretoja R., Isoaho H., Leino-Kilpi H. (2004). Nurse competence scale: Development and psychometric testing. J. Adv. Nurs..

[11] Nilsson J., Johansson E., Egmar A.C., Florin J., Leksell J., Lepp M.I., Gardulf A. (2014). Development and validation of a new tool measuring nurses self-reported competence: The nurse professional competence (NPC) scale. Nurse Educ. Today.

[12] Cowan N., Naveh-Benjamin M., Kilb A., Saults J.S. (2006). Life-span development of visual working memory: When is feature binding difficult?. Dev. Psychol..

[13] Short E.C. (1984). Competence reexamined. Educ. Theory.

[14] Black J., Allen D., Redfern L., Muzio L., Rushowick B., Balaski B., Martens P., Crawford N., Conlin-Saindon K., Chapman L. (2008). Competencies in the context of entry-level registered nurse practice: A collaborative project in Canada. Int. Nurs. Rev..

[15] Okumura M., Ishigaki T., Mori K., Fujiwara Y. (2022). Personality traits affect critical care nursing competence: A multicentre cross-sectional study. Intensive Crit. Care. Nurs..

[16] Blažun H., Kokol P., Vošner J. (2015). Survey on specific nursing competences: Students’ perceptions. Nurse Educ. Pract..

[17] Foss G.F., Janken J.K., Langford D.R., Patton M.M. (2004). Using professional specialty competencies to guide course development. J. Nurs. Educ..

[18] Newble D., Dawson B., Dauphinee D., Page G., Macdonald M., Swanson D., Mulholland H., Thomson A., van der Vleuten C. (1994). Guidelines for assessing clinical competence. Teach. Learn. Med. Int. J..

[19] Calzone K.A., Jenkins J., Masny A. (2002). Core competencies in cancer genetics for advanced practice oncology nurses. Oncol. Nurs. Forum.

[20] American Nurses Association Nursing: Scope and Standards of Practice. https://www.nursingworld.org/~4adb8c/globalassets/catalog/book-toc/toc-nursing-scope-and-standards-of-practice-3e.pdf.

[21] Hale N., Kirwan G., Manley K., McBride L. (2012). Core knowledge and skills framework dimensions. Integrated Core Career and Competence Framework for Registered Nurses.

[22] McMullan M., Endacott R., Scholes J., Gray M., Jasper M., Miller C., Webb C. (2003). Portfolios and assessment of competence: A review of the literature. J. Adv. Nurs..

[23] Mousa A. (2017). Nurse Staffing, Patient Falls and Medication Errors in Western Australian Hospitals: Is There a Relationship?. Ph.D. Dissertation.

[24] Hengstberger-Sims C., Cowin L., Eagar S., Cert E., Gregory L., Andrew S., Rolley J. (2008). Relating new graduate nurse competence to frequency of use. Collegian.

[25] Yang F.Y., Zhao R.R., Liu Y.S., Wu Y., Jin N.N., Li R.Y., Shi S.P., Shao Y.Y., Guo M., Arthur D. (2013). A core competency model for Chinese baccalaureate nursing graduates: A descriptive correlational study in Beijing. Nurse Educ. Today.

[26] Beyea S.C., Von Reyn L.K., Slattery M.J. (2007). A nurse residency program for competence development using human patient simulation. J. Nurs. Staff Dev..

[27] Burns P., Poster E.C. (2008). Competency development in new registered nurse graduates: Closing the gap between education and practice. J. Contin. Educ. Nurs..

[28] Marshburn D.M., Engelke M.K., Swanson M.S. (2009). Relationships of new nurses’ perceptions and measured performance-based clinical competence. J. Contin. Educ. Nurs..

[29] Piggott H. (2001). Facing reality: The transition from student to graduate nurse. Aust. Nurs. J..

[30] Chen Q., Liu D., Zhou C., Tang S. (2020). Relationship between critical thinking disposition and research competence among clinical nurses: A cross-sectional study. J. Clin. Nurs..

[31] Chen F.F., Chen S.Y., Pai H.C. (2019). Self-reflection and critical thinking: The influence of professional qualifications on registered nurses. Contemp. Nurse.

[32] Bagheri A., Akbari M. (2018). The impact of entrepreneurial leadership on nurses’ innovation behavior. J. Nurs. Scholarsh..

[33] Salamonson Y., Glew P., Everett B., Woodmass J.M., Lynch J., Ramjan L.M. (2019). Language support improves oral communication skills of undergraduate nursing students: A 6-month follow-up survey. Nurse Educ. Today.

[34] Missen K., McKenna L., Beauchamp A., Larkins J.A. (2016). Qualified nurses’ perceptions of nursing graduates’ abilities vary according to specific demographic and clinical characteristics. A descriptive quantitative study. Nurse Educ. Today.

[35] Boamah S.A., Laschinger H. (2016). The influence of areas of worklife fit and work-life interference on burnout and turnover intentions among new graduate nurses. J. Nurs. Manag..

[36] Caliskan A., Ergun Y.A. (2012). Examining job satisfaction burnout and reality shock amongst newly graduated nurses. Procedia-Soc. Behav. Sci..

[37] Dyess S.M., Sherman R.O. (2009). The first year of practice: New graduate nurses’ transition and learning needs. J. Contin. Educ. Nurs..

[38] Kramer M., Brewer B.B., Maguire P. (2013). Impact of healthy work environments on new graduate nurses’ environmental reality shock. West. J. Nurs. Res..

[39] Lofmark A., Smide B., Wikblad K. (2006). Competence of newly-graduated nurses: A comparison of the perceptions of qualified nurses and students. J. Adv. Nurs..

[40] Widarsson M., Asp M., Letterstål A., Källestedt M.L.S. (2020). Newly graduated Swedish nurses’ inadequacy in developing professional competence. J. Contin. Educ. Nurs..

[41] Kramer M. (1974). Reality Shock: Why Nurses Leave Nursing.

[42] Fagermoen M.S. (1997). Professional identity: Values embedded in meaningful nursing practice. J. Adv. Nurs..

[43] Heslop L., McIntyre M., Ives G. (2001). Undergraduate student nurses’ expectations and their self-reported preparedness for the graduate year role. J. Adv. Nurs..

[44] Knecht J.G., Fischer B. (2015). Undergraduate nursing students’ experience of service-learning: A phenomenological study. J. Nurs. Educ..

