# Supplementary material for: Assessment of Differential Perceptions of Core Nursing Competencies between Nurse Managers and Nursing Graduates: A Cross-Sectional Study
Source: Nurs Rep. 2023 Dec 18;13(4):1751–60. doi: 10.3390/nursrep13040145 (PMC10746040; doi:10.3390/nursrep13040145)
Supplement: Supplementary file 1 [file nursrep-13-00145-s001.zip › nursrep-2659178-supplementary.pdf]

Table S1: Scales for evaluating study variables in factor analysis

	Items	Cronbach's Alpha
	**Factor 1: Research Professionalism**	
**1**	Design nursing research of a high standard	**0.844**
**2**	Conduct research to solve clinical problems	
**3**	Aware of the trends and issues in nursing research	
**4**	Use research findings to inform nursing practice	
**5**	Have management skills	
**6**	Supervise newly enrolled nurses effectively	
**7**	Contribute to the application of new techniques	
**8**	Be able to communicate in English (reading and writing)	
**9**	Coach nursing students as needed	
**10**	Search and retrieve information through electronic databases	
**11**	Serve as a coordinator among the healthcare team members	
**12**	Improve standards of care based on new techniques	
	**Factor 2: Direct Treatment**	
**13**	Undertake basic nursing skills safely and confidently	**0.877**
**14**	Carry out medical orders correctly	
**15**	Being aware of and sensitive to changes in the patient's condition and notify doctors in time as needed	
**16**	Effectively perform basic life support skills	
**17**	Perform overall care for critically ill patients	
**18**	Provide nursing care according to standard procedure	
**19**	Make ethically and legally appropriate decisions	
**20**	Evaluate the effects of nursing interventions	
**21**	Revise nursing care plans based on the assessment data and provide individualized care	
**22**	Provide suggestions about patients' treatment programs from a nursing perspective	
	**Factor 3: Support and Communication**	
**23**	Provide psychological support	**0.853**
**24**	Educate patients about healthy lifestyle and help them to change unhealthy behaviors	
**25**	Provide education to patients about health promotion and disease prevention	
**26**	Gain support from patients' family and friends to facilitate patient's recovery	
**27**	Provide patients with a comfortable environment	
**28**	Be concerned and supportive when caring for patients	
**29**	Facilitate patients in getting support and resources from social organizations	
**30**	Communicate effectively with patients and families	
	**Factor 4: Professional Knowledge**	
**31**	Explain to patients the meaning of various diagnostic test results	**0.855**
**32**	Explain to patients the meaning of routine blood and urine tests	
**33**	Perform physical examination of patients	
**34**	Know the treatments for common diseases	
**35**	Evaluate the effects of medical treatments	
**36**	Make nursing diagnoses	
**37**	Respond effectively in rapidly changing situations	
	**Factor 5: Personal Abilities**	

**38**	Respond confidently and swiftly, remaining composed in every situation	**0.822**
**39**	Express self with aptness or propriety	
**40**	Decent appearance and gracious demeanor	
**41**	Emotional self-control	
**42**	Highly accountable and sympathetic when caring for patients	
**43**	Have keen insight	
**44**	Comply strictly with the regulations and standards of care at any time	
	**Factor 6: Critical Thinking and Innovation**	
**45**	Be able to analyze and explore problems with an open mind and creativity	**0.733**
**46**	Recognize the need and undertake professional self-development to update oneself with new information and techniques	
**47**	Consider feedback from colleagues for critical reflection on nursing practice	
